# Mechanism of Ginsenoside Rg_1_ in Regulating the Metabolic Function of Intestinal Flora for the Treatment of High-Purine Dietary Hyperuricemia

**DOI:** 10.3390/nu17111844

**Published:** 2025-05-28

**Authors:** Qiang Sun, Zhiman Li, Yang Yu, Yinshi Sun

**Affiliations:** 1 College of Chinese Medicinal Materials, Jilin Agricultural University, Changchun 130118, China; sunqiang133543@163.com; 2 Institute of Special Animal and Plant Sciences, Chinese Academy of Agricultural Sciences, Changchun 130112, China or lizm@caas.cn (Z.L.); 15941105393@163.com (Y.Y.)

**Keywords:** ginsenoside Rg_1_, hyperuricemia, inflammatory factors, gut microbiota, short-chain fatty acid

## Abstract

**Objective:** Study the mechanism of ginsenoside Rg_1_ in ameliorating hyperuricemia (HUA) induced by high-purine diet. **Methods**: Rats were randomly divided into groups, and the HUA model was established by administering a high-purine diet containing potassium oxonate combined with yeast. After the experiment, blood was collected via cardiac puncture, and the organ indices of the rats were calculated. Serum biochemical markers including aspartate aminotransferase (AST), alanine aminotransferase (ALT), triglyceride (TG), total cholesterol (TC), xanthine oxidase (XOD), creatinine (CREA), uric acid (UA), and blood urea nitrogen (BUN) were measured. Histopathological sections of the kidney and intestine were prepared. Western blot was used to assess the expression levels of intestinal occludin and zonula occludens-1 barrier proteins and key proteins in IL-17/NF-κB inflammatory pathways. After the experiment, fecal samples were collected from the rats. The gut microbiota of HUA-induced rats was analyzed via 16S rRNA sequencing, and the levels of short-chain fatty acids in the fecal samples were quantified using gas chromatography–mass spectrometry. **Results**: Ginsenoside Rg_1_ significantly increased body weight and organ indexes as well as reduced serum levels of BUN, CREA, ALT, AST, XOD, and UA. Pathologic analysis showed that ginsenoside Rg_1_ improved renal cell injury, glomerulosclerosis, and renal interstitial fibrosis while restoring intestinal barrier function. Ginsenoside Rg_1_ down-regulated the expression of inflammatory proteins and up-regulated the levels of intestinal barrier proteins. The results of 16S rRNA sequencing showed that ginsenoside Rg_1_ significantly increased the diversity index of gut microbiota and enhanced the number of beneficial bacteria in HUA rats. Short-chain fatty acids analysis demonstrated that ginsenoside Rg_1_ markedly elevated the levels of acetate, propionate, butyrate, and valerate in HUA rats. **Conclusions**: Ginsenoside Rg_1_ ameliorates and treats HUA by improving the composition of intestinal flora and inhibiting the IL-17/NF-κB signaling pathway to reduce inflammatory factors in the intestinal tract in HUA rats.

## 1. Introduction

With societal development, irregular living environments and dietary habits have led to a growing prevalence of hyperuricemia (HUA), which has become the world’s second most common metabolic disorder with an increasing trend among younger populations [1]. The pathogenesis of HUA primarily involves excessive uric acid production and impaired renal excretion. The uric acid production is primarily mediated by the enzymatic activity of xanthine dehydrogenase and xanthine oxidase [2,3]. The uricase is an enzyme that catalyzes the oxidation of uric acid into allantoin. However, humans and most mammals lack functional uricase activity due to evolutionary gene silencing. This deficiency impairs the metabolic clearance of uric acid, particularly when excessive purine-rich foods are consumed. Consequently, the inability to effectively oxidize and eliminate uric acid leads to its pathological accumulation, resulting in elevated serum urate levels. Uric acid is primarily eliminated through renal excretion and intestinal microbiota-mediated clearance. Dysfunction of either the kidneys or gut microbiota homeostasis can impair urate excretion, thereby contributing to the development of HUA. Approximately 70% of endogenously produced uric acid is excreted via renal metabolism, while the remaining 30% is cleared by gut microbiota-mediated degradation [4]. In terms of treatment, the intestinal flora has a great influence on the production and excretion of uric acid. From the point of view of the uric acid generation pathway, some studies have shown that Escherichia coli in the intestinal flora secretes xanthine oxidase, which promotes the elevation of uric acid [5]. From the perspective of the process of uric acid excretion, some relevant studies have also found that Lactobacillus in the intestinal flora can secrete uric acid oxidase [6], which promotes the decomposition of uric acid and accelerates the excretion of uric acid. Xu Y et al. found that IL-6, IL-17, and VEGFA are key targets in HUA through network pharmacology analysis, and drugs may reduce uric acid levels through signaling pathways such as IL-17 [7]. Currently, commonly used clinical drugs include allopurinol, benzbromarone, and febuxostat [8]. Although it exhibits certain therapeutic effects, this treatment is associated with significant adverse effects. In contrast, traditional Chinese medicine (TCM) plays an increasingly important role in the prevention and treatment of HUA by virtue of its low toxicity, high safety, and significant efficacy.

Panax ginseng C. A. Mey, a perennial herb of the Araliaceae family, has been used medicinally for thousands of years. Its earliest documentation appears in Shennong’s Classic of Materia Medica, which describes ginseng as sweet and slightly cold in nature, with primary functions including tonifying the five viscera, calming the spirit, stabilizing the soul, relieving palpitations, eliminating pathogenic factors, improving vision, enhancing intelligence, and prolonging life with long-term use [9]. As a fundamental component of traditional Chinese medicine, ginseng is widely applied in disease treatment, health maintenance, and clinical therapy. Ginsenosides, particularly ginsenoside Rg_1_, represent its major bioactive constituents and serve as key markers for evaluating ginseng quality and medicinal value [10]. Ginsenoside Rg_1_, a protopanaxatriol-type saponin isolated from ginseng, exhibits diverse pharmacological activities including antitumor [11], anti-fatigue [12], cardioprotective [13,14], neuroprotective [15,16], hepatoprotective [17,18], anti-aging, and renal protective effects [19,20,21]. Recent studies have confirmed its uric acid-lowering potential [21], yet the underlying mechanisms remain insufficiently elucidated.

Therefore, this study employed the HUA rat model induced by a purine-rich diet to investigate the effects of ginsenoside Rg_1_ on body weight changes, organ indices, serum uric acid levels and biochemical parameters, histopathological alterations in intestinal and renal tissues, modulation of the IL-17/NF-κB signaling pathway, expression of intestinal barrier proteins, as well as gut microbiota composition and SCFAs profiles, aiming to elucidate the underlying mechanisms and provide a theoretical foundation for ginsenoside Rg_1_ in the amelioration and treatment of HUA.

## 2. Materials and Methods

### 2.1. Experimental Animals

Forty male Sprague–Dawley rats (specific pathogen-free [SPF] grade, body weight 140–150 g) were purchased from Liaoning Changsheng Biotechnology Co., Ltd., Shenyang, China. (Quality certification provided by Suzhou Xishan Biotechnology Co., Ltd., Suzhou, China; Production License No.: SCXK [Liao] 2020-0001) and housed at the Institute of Special Animal and Plant Sciences of Chinese Academy of Agricultural Sciences.

### 2.2. Reagents and Instruments

Ginsenoside Rg_1_ (purity ≥99.9%) was purchased from Ruifengfan Technology Co., Ltd., Kaifeng, China. The hyperuricemic diet containing potassium oxonate combined with yeast was obtained from Beijing Botai Hongda Biotechnology Co., Ltd., Beijing, China. Biochemical assay kits including AST (C010-3-1), ALT (C009-3-1), TG (A110-1-1), TC (A111-1-1), XOD (A002-1-1), CREA (C011-2-1), UA (C012-2-1), and BUN (C013-2-1) were acquired from Nanjing Jiancheng Bioengineering Institute, Nanjing, China. RIPA lysis buffer was purchased from Bio-world, Dublin, OH, USA. Primary antibodies against GAPDH (ab8245), IL-17 (ab100702), IL-17RA (ab263908), IL-6 (ab233706), IKB-α (ab32518), TRAF6 (ab40675), NF-κB p65 (ab32536), and ZO-1 (ab221547), along with secondary antibodies including goat anti-rabbit IgG H&L (HRP) and goat anti-mouse IgG H&L (HRP), were obtained from Shanghai Abcam Trading Co., Ltd., Shanghai, China. Anti-Occludin antibody was purchased from Santa Cruz Biotechnology, Santa Cruz, CA, USA. ECL chemiluminescence substrate was acquired from Shanghai Yamei Biomedical Technology Co., Ltd., Shanghai, China. Protein-related reagents including prestained protein marker, skim milk powder, 5X SDS-PAGE loading buffer, and SDS-PAGE gel preparation kit were supplied by Wuhan Boster Biological Technology Co., Ltd., Wuhan, China. Histological reagents including 10% neutral buffered formalin (SL1560-500 mL) were purchased from Beijing Coolaber Technology Co., Ltd., Beijing, China. while the hematoxylin and eosin stain (G1005-100 mL) was obtained from Wuhan Servicebio Technology Co., Ltd., Wuhan, China, and neutral balsam was acquired from Solarbio Science & Technology Co., Ltd., Beijing, China.

Epoch2 microplate reader (BioTek Instruments, Winooski, VT, USA); MS204S electronic balance (Mettler Toledo, Greifensee, Switzerland); HH-4A digital constant temperature water bath (Changzhou Guohua Electric Appliance Co., Ltd., Changzhou, China); LDZM-40KCS-2 vertical pressure steam sterilizer (Shanghai Shen’an Medical Equipment Factory, Shanghai, China); DYY-8C electrophoresis power supply (Bio-Rad, Hercules, CA, USA); SF-TDL large-capacity centrifuge (Shanghai Feiqiao Analytical Instrument Co., Ltd., Shanghai, China); EG1150H tissue embedding machine and RM2255 microtome (Leica, Wetzlar, Germany); KD-P tissue floating bath (Jinhua Kedi Instrument Equipment Co., Ltd., Jinhua, China); IX53 fluorescence microscope (Beijing Xinaifang Medical Equipment Co., Ltd., Beijing, China); MV-100 vortex mixer (Wuhan Servicebio Technology Co., Ltd., Wuhan, China); Heraeus Megafuge 8R refrigerated centrifuge (Thermo Fisher Scientific, Waltham, MA, USA); WP-UP-WF-40 ultrapure water system (Sichuan Water Treatment Equipment Co., Ltd., Chengdu, China); and UV spectrophotometer were employed.

### 2.3. Animal Modeling and Drug Administration

Sprague–Dawley rats were acclimatized for 7 days under controlled environmental conditions (temperature: 22 ± 2 °C; relative humidity: 55 ± 5%) with free access to food and water. All experimental procedures strictly adhered to the Laboratory Animal Care and Use Guidelines of the Institute of Special Animal and Plant Sciences of Chinese Academy of Agricultural Sciences. Sample sizes were determined based on power analysis to ensure sufficient statistical power to detect differences between groups. Based on preliminary data, we assumed effect sizes and standard deviations for key outcome metrics (e.g., inflammatory cytokine levels), and we derived a minimum of 7 animals per group (calculated using Prism 8.0.1). To account for possible attrition (e.g., death or loss of skill), we increased the sample size to 8 rats per group (total *n* = 40), including normal control (NC), model (M), allopurinol (AP), ginsenoside Rg_1_ low-dose (Rg_1_-L), and ginsenoside Rg_1_ high-dose (Rg_1_-H). All groups except NC were fed with hyperuricemia-inducing diet (containing potassium oxonate and yeast extract), while all animals received autoclaved distilled water throughout the experiment. Body weights were recorded weekly during the feeding period. After 3 weeks of dietary induction, blood samples were collected via orbital venous plexus puncture and centrifuged at 3500 rpm (4 °C) to obtain serum. Successful model establishment was confirmed when serum uric acid levels in treated groups showed statistically significant elevation compared to the NC group.

Following successful model establishment, intragastric administration was initiated. The AP group received allopurinol (25 mg/kg), while Rg_1_-L and Rg_1_-H groups were administered ginsenoside Rg_1_ at 12.5 mg/kg and 25 mg/kg, respectively. Treatments were delivered daily for 8 consecutive weeks. At experimental termination, body weights were recorded after the final administration. Following a 24 h fasting period (water ad libitum), the rats were anesthetized and euthanized by blood sampling through the heart. The liver, lungs, kidneys, and spleen were excised and weighed, with renal and ileal tissues immediately fixed in 10% neutral buffered formalin for histopathological examination while additional tissue aliquots were snap-frozen in liquid nitrogen and stored at −80 °C for molecular analyses. Blood samples were allowed to clot for 30 min at room temperature before centrifugation at 4500 rpm (4 °C) to obtain serum which was aliquoted into sterile 1.5 mL microcentrifuge tubes. Fresh fecal samples were aseptically collected into cryovials and preserved at −80 °C for subsequent microbiome analysis. Experimental operators were unblinded to administer treatments, while outcome assessors and statisticians remained blinded through pseudonymized sample coding.

### 2.4. Assessment of Body Weight and Serum Biochemical Parameters in HUA Rats

Throughout the experimental period, body weight and food intake were measured weekly at fixed time points. Upon completion of the study, blood samples obtained via cardiac puncture were centrifuged at 4500 rpm for 10 min at 4 °C to isolate serum. The resulting supernatant was analyzed using assay kits to determine key biochemical parameters, including aspartate aminotransferase (AST), alanine aminotransferase (ALT), total cholesterol (TC), triglycerides (TG), creatinine (CREA), uric acid (UA), blood urea nitrogen (BUN), and xanthine oxidase (XOD) levels.

### 2.5. Histopathological Examination of Renal and Ileal Tissues

The collected kidney and ileum tissues were fixed in 10% neutral buffered formalin for 24–48 h, followed by sequential dehydration through graded ethanol series (70–100%) and xylene clearance. The dehydrated tissues were embedded in paraffin and sectioned at 5 μm thickness using a rotary microtome. Tissue sections were floated on a warm water bath (40–45 °C) to ensure complete expansion, then mounted onto glass slides. After deparaffinization with xylene and rehydration through descending alcohol concentrations (70–100%), the sections were stained with hematoxylin and eosin (H&E) using standard protocols. Finally, the stained sections were cover-slipped with 10% neutral balsam mounting medium. Histomorphological evaluation was performed under light microscopy at 40× and 100× magnifications, with particular attention to glomerular morphology, tubular integrity, interstitial inflammation, villus architecture, and epithelial barrier continuity.

### 2.6. WB Analysis of Inflammatory Pathway and Intestinal Barrier Proteins

The appropriate amount of tissue was added to the lysis solution and fully lysed to extract the supernatant, and the corresponding concentration and protein sampling volume were calculated. The extracted supernatant was mixed with protein sampling buffer and put into a metal bath for protein denaturation. The prepared sample was added into the separation gel, and electrophoresis was carried out at a constant voltage of 80 V for about 30 min until the protein sample was at the boundary between the concentration gel and the separation gel, and then changed to a constant voltage of 120 V for about 1.5 h to the bottom of the gel. The gel and the PVDF membrane were put together in the membrane transfer buffer in the order of white plate–sponge pad–filter paper–PVDF membrane–gel–filter paper–sponge pad–black plate and then the membrane was transferred at a constant voltage of 70 V. The time of transferring the membrane was determined according to the molecular weight of the protein. After the membrane transfer was completed, the membrane was closed with 10% skimmed milk powder and rinsed with PBST. The PVDF membrane was immersed in primary antibody and incubated in the refrigerator at 4 °C overnight, and the corresponding secondary antibody was incubated at room temperature for 2 h. The enhancement solution and the peroxidase solution in the ECL reagent were mixed at a ratio of 1:1 so as to make the developer solution sufficiently and uniformly cover the PVDF membrane. It was then put into the imager for development.

### 2.7. 16S rRNA Microbial Community Sequencing

Fecal samples were aseptically collected from each group of rats and sent to Beijing Novogene Bioinformatics Technology Co., Ltd., Beijing China, for intestinal microbiota analysis using 16S rRNA sequencing. Genomic DNA was extracted from the fecal samples using a soil DNA extraction kit. The NEB Next Ultra II DNA Library Prep Kit (Cat. No. E7645B, Ipswich, MA, USA) was employed for library construction, followed by sequencing on the NovaSeq 6000 platform. PCR amplification was performed targeting the V3 + V4 hypervariable regions of 16S rRNA using specific primers: 341F (CCTAYGGGRBGCASCAG) and 806R (GGACTACNNGGGTATCTAAT). A short-fragment library was constructed based on the characteristics of the amplified regions, and paired-end sequencing was conducted using the Illumina NovaSeq platform. After read splicing and filtering, OTUs (Operational Taxonomic Units) clustering or ASVs (Amplicon Sequence Variants) noise reduction, the resulting valid data were subsequently subjected to species annotation as well as species composition analysis, alpha diversity analysis, beta diversity analysis, and Lefse analysis.

### 2.8. Determination of Short-Chain Fatty Acids (SCFAs)

Prior to the end of the experiment, an adequate amount of rat feces was aseptically collected into sterile cryotubes under sterile conditions. The fecal samples were homogenized with ultrapure water at a 1:7 (*w*/*v*) ratio using a homogenizer (Bertin Technologies, Montigny-le-Bretonneux, France) for 30 s, followed by vortex mixing. After cold extraction at 4 °C for 12 h, the samples were centrifuged at 3000 rpm for 10 min. The supernatant was collected and further centrifuged at 15,000 rpm for 10 min. The resulting supernatant was mixed with 2-ethylbutyric acid (2EB) metaphosphoric acid solution, vortexed thoroughly, and placed on ice for 30 min. Following another centrifugation at 15,000 rpm for 10 min at 4 °C, the supernatant was filtered through a membrane into vials for analysis. The concentrations of SCFAs in rat feces, including acetic acid, propionic acid, butyric acid, valeric acid, hexanoic acid, isobutyric acid, and isovaleric acid, were determined using gas chromatography–mass spectrometry (GC-MS, Santa Clara, CA, USA). The SCFA concentrations were quantified and expressed in mmol/L.

### 2.9. Data Processing

GraphPad Prism 8.0.1 software was used to process and analyze the experimental data, and independent sample *t*-test was used for comparison between 2 groups. One-way ANOVA was used for comparison between multiple groups, and the difference was considered to be statistically significant at *p* < 0.05.

## 3. Results

### 3.1. Effect of Ginsenoside Rg_1_ on Body Weight and Organ Indices in HUA Rats

**Figure 1:**
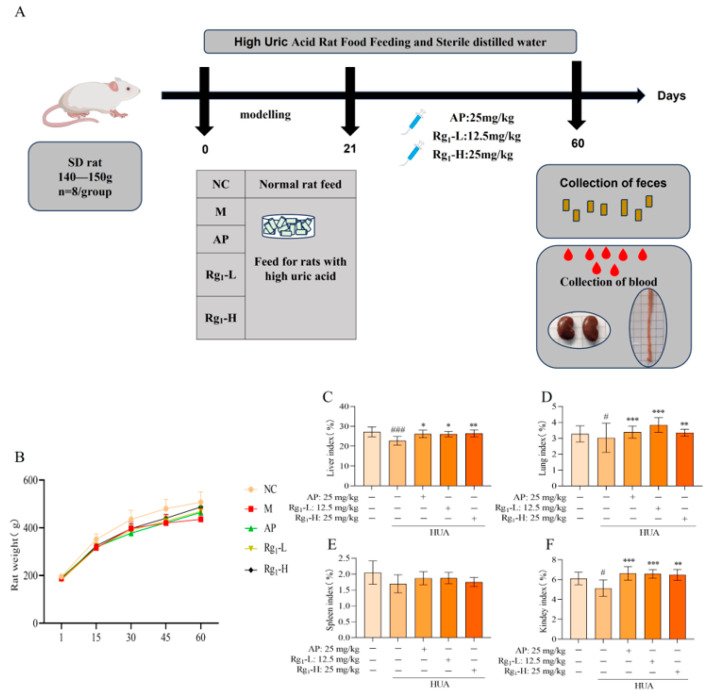
Experimental design protocol and effects of ginsenoside Rg_1_ on body weight and organ index in HUA rats. (**A**) Experimental design program; (**B**) Effect of ginsenoside Rg_1_ on body weight of HUA rats; (**C**) Liver index; (**D**) Lung index; (**E**) Spleen index; (**F**) Kidney index. ^###^ *p* < 0.001, ^#^ *p* < 0.05 vs. normal group, *** *p* < 0.001, ** *p* < 0.01, * *p* < 0.05 vs. model group. “+” indicates drug delivery intervention. “−” indicates no intervention.

Body weight serves as a fundamental parameter for assessing animal health status, while organ indices reflect the impact of pharmacological interventions on specific organs, collectively indicating the overall physiological influence of treatment. As illustrated in Figure 1B, during the modeling period, all groups exhibited normal behavior, except for the M group, which showed dull fur, reduced food intake, and slower weight gain compared to the NC group. During the treatment phase, both the AP and ginsenoside Rg_1_-administered groups demonstrated improved fur texture and accelerated weight gain relative to the M group. As illustrated in Figure 1C–F, terminal analyses revealed that compared to the NC group, the M group exhibited statistically significant reductions in liver (*p* = 0.0008), lung (*p* = 0.0450), and kidney indices (*p* = 0.0330), with a non-significant decrease in spleen index. In contrast, the Rg_1_-L groups showed significant increases in liver (*p* = 0.0221), kidney (*p* = 0.001), and lung indices (*p* = 0.0008) compared to the M group; Rg_1_-H groups showed significant increases in liver (*p* = 0.0058), kidney (*p* = 0.0017), and lung indices (*p* = 0.0084) while spleen index remained unchanged. These results demonstrate that ginsenoside Rg_1_ effectively maintains body weight and enhances organ indices (kidney, liver, and lung) in HUA rats.

### 3.2. Effects of Ginsenoside Rg_1_ on Biochemical Parameters and Renal, Intestinal Histopathology in HUA Rats

**Figure 2:**
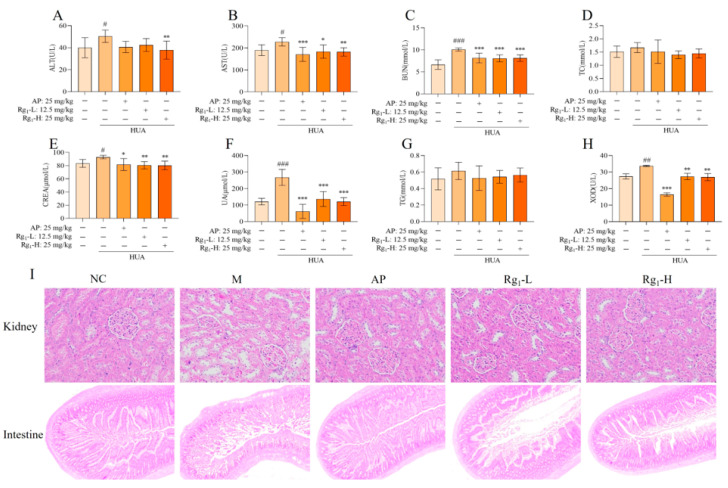
Effects of ginsenoside Rg_1_ on biochemical parameters and renal, intestinal histopathology in HUA rats. (**A**) ALT content; (**B**) AST content; (**C**) BUN content; (**D**) TC content; (**E**) CERA content; (**F**) UA content; (**G**) TG content; (**H**) XOD content; (**I**) Kidney pathology and tissue effects (200×) and intestinal pathology and tissue effects (40×). ^###^ *p* < 0.001, ^##^ *p* < 0.01, ^#^ *p* < 0.05 vs. normal group, *** *p* < 0.001, ** *p* < 0.01 vs., * *p* < 0.05 vs. model group. “+” indicates drug delivery intervention. “−” indicates no intervention.

The etiology of HUA is diverse, and abnormal uric acid synthesis or excretion can occur due to kidney diseases, metabolic disorders, or drug effects. Consequently, during the pathophysiological progression of HUA, serum biochemical indicators such as TG, TC, AST, ALT, CERA, UA, and BUN may exhibit abnormalities [22]. As shown in Figure 2A–H, compared with the NC group, the M group demonstrated significantly higher expression levels of AST (*p* = 0.0389), ALT (*p* = 0.0377), BUN (*p* = 0.0001), CERA (*p* = 0.0326), UA (*p* = 0.0001), and XOD (*p* = 0.0039). Additionally, TG and TC levels in the M group showed an increasing trend compared to the NC group, though without statistical significance. In contrast, the Rg_1_-L groups exhibited significantly lower expression levels of AST (*p* = 0.0140), BUN (*p* = 0.0007), CERA (*p* = 0.0049), UA (*p* = 0.0001), and XOD (*p* = 0.0036) compared to the M group. Also, the Rg_1_-H groups exhibited significantly lower expression levels of AST (*p* = 0.0058), BUN (*p* = 0.0006), CERA (*p* = 0.0027), UA (*p* = 0.0001), XOD (*p* = 0.0024), and ALT (*p* = 0.0083). Furthermore, TG and TC levels displayed a decreasing trend relative to the M group, though not statistically significant.

Histopathological examination provides direct visualization of organ lesions and the extent of tissue damage, playing a crucial role in investigating drug mechanisms and evaluating therapeutic efficacy and safety. As illustrated in Figure 2I, compared with the NC group, the M group exhibited loosely arranged, irregular, and structurally disrupted renal tissues, with marked glomerular sclerosis and atrophy, severe disorganization and vacuolar degeneration of renal tubular epithelial cells, pronounced interstitial fibrosis, and significant inflammatory cell infiltration. Compared with the M group, the AP group and ginsenoside Rg_1_-treated groups exhibited tightly arranged and well-organized renal tissue structure, with no significant glomerular sclerosis and normal morphology. The renal tubular epithelial cells displayed orderly alignment, and the renal interstitium showed no evident fibrosis or inflammatory cell infiltration. As shown in Figure 2I, compared with the NC group, the M group demonstrated markedly shortened intestinal villi with severe structural damage, disorganized mucosal epithelial cell arrangement, shallow crypts, irregular outer membrane layers, and inflammatory cell infiltration in the lamina propria. These observations indicate substantial intestinal tissue destruction and severe impairment of the intestinal barrier. In contrast, the AP group and ginsenoside Rg_1_-treated groups exhibited elongated villi, deepened crypts, regular and well-organized outer membrane layers, and neatly arranged mucosal epithelial cells, with the ileal tissue structure of gavaged rats approximating normal morphology.

### 3.3. Effects of Ginsenoside Rg_1_ on IL-17/NF-κB Pathway and Intestinal Barrier Proteins in HUA Rat Intestinal Tissues

**Figure 3:**
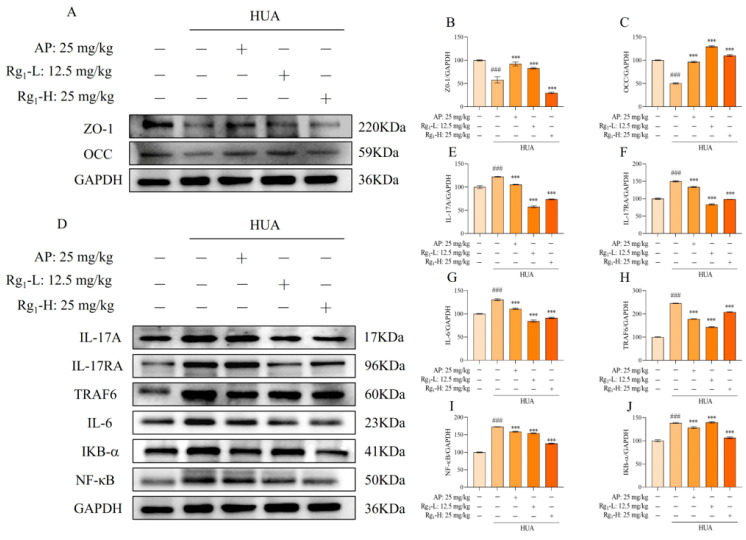
Effects of ginsenoside Rg_1_ on IL-17/NF-κB pathway and intestinal barrier proteins in rat intestinal tissues. (**A**) Representative bands of intestinal barrier protein; (**B**) Protein expression of ZO-1; (**C**) Protein expression of OCC; (**D**) Representative bands of IL-17/NF-κB pathway; (**E**) Protein expression of IL-17A; (**F**) Protein expression of IL-17RA; (**G**) Protein expression of IL-6; (**H**) Protein expression of TRAF6; (**I**) Protein expression of NF-κB; (**J**) Protein expression of IKB-α. ^###^ *p* < 0.001 vs. normal group, *** *p* < 0.001 vs. model group. “+” indicates drug delivery intervention. “−” indicates no intervention.

The IL-17/NF-κB signaling pathway represents a crucial intracellular signaling cascade that plays a significant role in inflammatory responses and cancer development. As shown in Figure 3D–J, compared with the NC group, the M group exhibited significantly elevated protein expression levels of IL-17A, IL-17RA, TRAF6, IL-6, IKB-α, and NF-κB (*p* = 0.0001), indicating the onset of inflammatory responses. Following ginsenoside Rg_1_ intervention, the expression levels of these inflammatory mediators (IL-17, IL-17RA, TRAF6, IL-6, IKB-α, and NF-κB) were markedly reduced and approached those observed in the NC group.

Intestinal barrier proteins, primarily localized in intestinal epithelial cells, serve critical functions in maintaining intestinal integrity, stabilizing the intestinal microenvironment, facilitating nutrient absorption, reducing inflammatory cell infiltration, and preserving gut microbiota balance. Figure 3A–C demonstrates that compared with the NC group, the M group showed significantly decreased expression of intestinal barrier proteins (ZO-1 and OCC) accompanied by increased intestinal permeability (*p* = 0.0001). Ginsenoside Rg_1_ treatment significantly upregulated the expression levels of ZO-1 and OCC in the intervention groups (*p* = 0.0001). These findings collectively demonstrate that ginsenoside Rg_1_ not only effectively reduces inflammatory factors in rat intestinal tissues, thereby preserving intestinal function, but also enhances intestinal barrier protein expression to protect intestinal barrier integrity and maintain intestinal homeostasis.

### 3.4. Effects of Ginsenoside Rg_1_ on Gut Microbiota α-Diversity, β-Diversity, and OTU Profiles in HUA Rats

**Figure 4:**
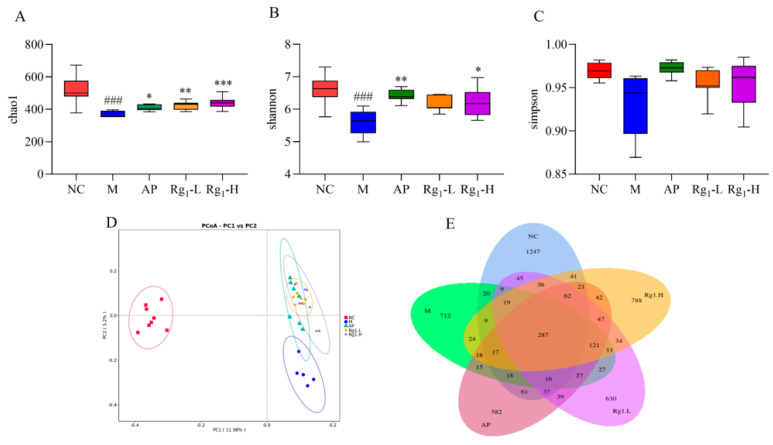
Effects of ginsenoside Rg_1_ on gut microbiota α-diversity, β-diversity, and OTU profiles in HUA rats. (**A**) Chao1 index analysis; (**B**) Shannon index analysis; (**C**) Simpson index analysis; (**D**) β-Diversity analysis; (**E**) Quantitative analysis of OTUs of intestinal flora. ^###^ *p* < 0.001 vs. normal group, *** *p* < 0.001, ** *p* < 0.01, * *p* < 0.05 vs. model group.

To further investigate the potential therapeutic mechanisms of ginsenoside Rg_1_ against HUA, we employed 16S rRNA sequencing technology to systematically analyze its effects on the gut microbiota composition in HUA rats. Alpha diversity analysis incorporated the Chao1 index, Shannon index, and Simpson index, which reflect, respectively, the total species richness, overall community diversity, and evenness of species distribution within microbial populations. Higher index values indicate greater total species richness, increased microbial diversity, and more balanced species distribution within the community. As shown in Figure 4A–C, compared to the NC group, the M group exhibited a significant decrease in the Chao1 index (*p* = 0.0001). In contrast, the Rg_1_-L group (*p* = 0.0064) and the Rg_1_-H group (*p* = 0.0202) showed marked increases relative to the M group. Similarly, the Shannon index was significantly reduced in the M group versus NC controls (*p* = 0.0003), while the ginsenoside Rg_1_-H groups demonstrated elevated indices compared to the M group (*p* = 0.0459). Compared to the NC group, the M group showed decreased Simpson index values, while both the AP group and ginsenoside Rg_1_-treated groups exhibited increasing trends in Simpson indices relative to the M group, though these differences did not reach statistical significance.

Beta diversity analysis refers to the assessment of microbial community differentiation among distinct individuals or ecological populations, which holds critical significance for investigating the structural and functional characteristics of gut microbiota. As demonstrated in Figure 4D, the substantial separation between the NC and M groups along the principal coordinates indicates significant inter-group differences in microbial composition. Notably, compared to the M group, both the AP group and ginsenoside Rg_1_-treated groups exhibited a directional shift toward the NC group along the PC2 axis. These findings suggest that ginsenoside Rg_1_ effectively reduces beta-diversity disparities and ameliorates gut microbiota dysbiosis in HUA rats.

OTU analysis involves clustering microorganisms with identical molecular signatures, enabling efficient comparison of inter-sample variations and shared features among microbial communities. This approach is fundamentally important for elucidating the compositional and functional attributes of gut microbiota. Following sequencing of rat fecal samples using the NovaSeq platform, the obtained sequences were assembled and clustered into OTUs. Subsequent bioinformatic processing using R software (R 4.4.0) quantified OTU distributions across experimental groups, with Venn diagrams visually representing both shared and unique OTUs among different samples or groups. As shown in Figure 4E, the NC, M, AP, Rg_1_-L, and Rg_1_-H groups exhibited 1254, 473, 524, 638, and 796 unique OTUs, respectively, with 264 OTUs shared across all groups. Considerable divergence in microbial composition was observed between the NC group and other experimental groups. Following ginsenoside Rg_1_ intervention, both Rg_1_-treated groups (Rg_1_-L and Rg_1_-H) demonstrated increased OTU numbers, indicating that ginsenoside Rg_1_ effectively restores gut microbial balance in HUA rats.

### 3.5. Analysis of Gut Microbiota Composition and LEfSe in HUA Rats Treated with Ginsenoside Rg_1_

**Figure 5:**
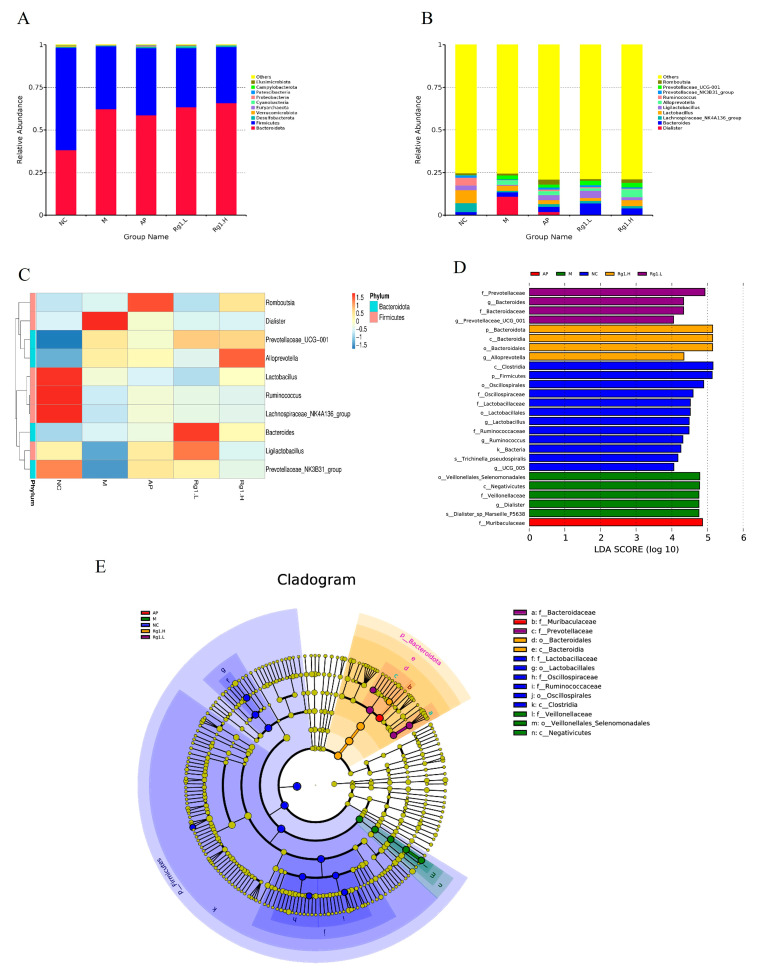
Species composition analysis and LEfSe analysis of ginsenoside Rg_1_ on intestinal flora of HUA rats. (**A**) Analysis of species composition at the gate level; (**B**) Analysis of species composition at the genus level; (**C**) Heat map of species composition at genus level; (**D**) Histogram of the distribution of LDA values of the dominant biomarker taxa in each group; (**E**) Taxonomic branching diagram of the dominant biomarker taxa in each group.

To further investigate differences in microbial composition among samples, we performed taxonomic composition analysis using abundance data and heatmap visualization of the top 10 most abundant bacterial species based on mean abundance values. Following 16S rRNA sequencing of fecal samples from each experimental group, we characterized the gut microbiota structural composition at multiple taxonomic levels. At the phylum level, the predominant bacterial taxa in fecal samples across all experimental groups primarily included *Firmicutes*, *Bacteroidota*, *Desulfobacterota*, *Euryarchaeota,* etc. As demonstrated in Figure 5A, compared to the NC group, the M group exhibited increased relative abundances of *Bacteroidota* and *Euryarchaeota*, along with decreased abundances of *Firmicutes* and *Desulfobacterota*. Compared to the M group, ginsenoside Rg_1_-treated groups showed increased relative abundances of *Bacteroidota*, *Desulfobacterota*, and *Euryarchaeota*, along with decreased abundance of *Firmicutes*.

At the genus level, Figure 5B,C reveals that compared to the NC group, the M group exhibited a significant increase in *Dialister* abundance while showing marked reductions in *Lachnospiraceae_NK4A136_group*, *Ligilactobacillus*, and *Lactobacillus*. In comparison with the M group, both the AP group and ginsenoside Rg_1_-treated groups demonstrated significantly increased abundances of *Ligilactobacillus*, *Lactobacillus*, *Ruminococcus*, and *Lachnospiraceae_NK4A136_group*. These findings demonstrate that ginsenoside Rg_1_ effectively ameliorates HUA-induced gut microbiota dysbiosis by selectively enriching beneficial bacterial taxa while reducing pathogenic populations, thereby restoring microbial homeostasis to alleviate HUA progression.

To better analyze biological relevance and statistical significance while identifying statistically differential biomarkers between groups, we performed LEfSe (Linear Discriminant Analysis Effect Size) analysis to systematically explore potential microbial biomarkers exhibiting significant inter-group variations across all experimental cohorts. As shown in Figure 5D,E, several taxa including *Clostridia*, *Firmicutes*, *Oscillospirales*, *Lactobacillus*, *UCG-005*, and *Bacteria* exhibited significant compositional influences on the NC group. The M group was predominantly characterized by potentially pathogenic taxa including *Veillonellales*_*Selenomonadales*, *Negativicutes*, *Dialister*, and *Dialisteria_sp_Marseille_P5638*, while ginsenoside Rg_1_-treated groups showed enrichment of beneficial microbiota such as *Prevotellaceae*, *Prevotellaceae_UCG-001*, *Bacteroidota*, *Bacteroidaceae*, and *Alloprevotella*. These results demonstrate that ginsenoside Rg_1_ intervention effectively restores gut microbial homeostasis in HUA rats by selectively increasing beneficial bacterial populations while reducing pathogenic taxa.

### 3.6. Effects of Ginsenoside Rg_1_ on SCFA Levels in the Intestine of HUA Rats

**Figure 6:**
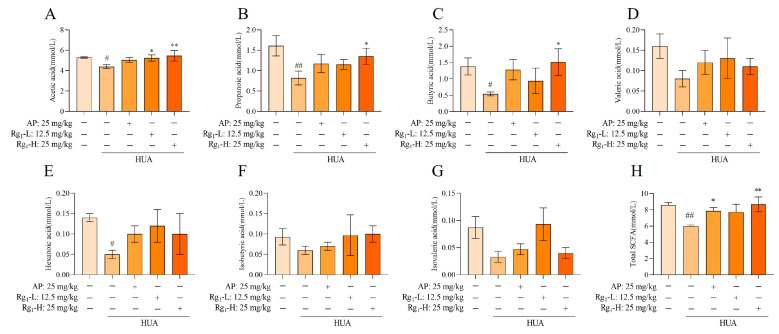
Effect of ginsenoside Rg_1_ intervention on the content of SCFAs. (**A**) Acetic acid content; (**B**) Propionic acid content; (**C**) Butyric acid content; (**D**) Valeric acid content; (**E**) Hexanoic acid content; (**F**) Isobutyric acid content; (**G**) Isovaleric acid content; (**H**) Total SCFAs content. ^##^ *p* < 0.01, ^#^ *p* < 0.05 vs. normal group, ** *p* < 0.01, * *p* < 0.05 vs. model group. “+” indicates drug delivery intervention. “−” indicates no intervention.

SCFAs are a class of organic acids produced by gut microbiota, particularly beneficial bacterial species, through fermentation processes. These compounds play crucial roles in maintaining intestinal homeostasis and regulating systemic physiological functions. Alterations in SCFA concentrations can serve as indirect indicators of both compositional and functional changes in the gut microbiota. As shown in Figure 6A–H, compared with the NC group, the M group exhibited significantly reduced levels of acetate (*p* = 0.0259), propionate (*p* = 0.0043), butyrate (*p* = 0.0497), and hexanoate (*p* = 0.0312), while valerate, isovalerate, and isobutyrate showed decreasing trends without statistical significance. Following ginsenoside Rg_1_ intervention, both the acetate level (*p* = 0.0356) in the Rg_1_-L group and the acetate (*p* = 0.0096), propionate (*p* = 0.0498), and butyrate (*p* = 0.0226) levels in the Rg_1_-H group showed significant increases. Although the remaining treatment groups demonstrated upward trends in SCFA levels, these changes did not reach statistical significance. Notably, ginsenoside Rg_1_ intervention significantly increased the total SCFA concentrations across all treatment groups (*p* = 0.0035). In summary, ginsenoside Rg_1_ intervention promoted the production of all seven measured SCFAs and significantly elevated total SCFA levels, indicating its potent modulatory effects on the metabolic functionality of gut microbiota.

## 4. Discussion

HUA is a chronic, multifactorial, and complex disorder primarily associated with decreased uric acid excretion and overproduction in the body [23,24,25]. Ginsenoside Rg_1_ exhibits diverse pharmacological effects, including neuroprotective, cardioprotective, immunomodulatory, anti-fatigue, anti-inflammatory, vasodilatory, hypolipidemic, and anti-diabetic properties [26,27,28,29,30]. However, studies investigating the potential of ginsenoside Rg_1_ to reduce uric acid levels and ameliorate HUA remain limited. Fan [21] demonstrated that ginsenoside Rg_1_ possesses uric acid-lowering effects, although the underlying mechanisms remain unclear. Therefore, the purpose of this experiment was to investigate the mechanism of action of ginsenoside Rg_1_ in improving HUA. This study established the HUA rat model by administering a high-purine diet containing potassium oxonate combined with yeast. Potassium oxonate specifically inhibits uricase activity, thereby blocking the uric acid metabolic pathway, while the purines in yeast are catalyzed by xanthine oxidase to generate uric acid. The synergistic effect of these two interventions significantly elevated uric acid levels in rats, successfully inducing the HUA model. The results of the study show that ginsenoside Rg_1_ treatment in HUA rats effectively maintained body weight, increased organ indices of the lungs, kidneys, and liver, and significantly reduced serum biochemical parameters including TG, TC, AST, ALT, CERA, UA, and BUN. Histopathological examination revealed that ginsenoside Rg_1_ intervention restored renal tissue damage and preserved intestinal functional integrity. Current evidence suggests that IL-6 protein serves as a core target through which gut microbiota metabolites influence HUA, and that the IL-17 pathway plays a crucial role in gut microbiota-mediated HUA treatment [31]. IL-17A serves as the upstream regulator of the IL-17 pathway, while IL-6 acts as its downstream target, both playing pivotal roles in various biological processes. Moreover, TRAF6—an intermediate signaling molecule in the IL-17 pathway—modulates the NF-κB signaling pathway, thereby exerting broad regulatory effects on the overall biological process. In our study, ginsenoside Rg_1_ intervention significantly reduced the expression of the upstream factor IL-17A, suppressed TRAF6 and IL-6 expression, and downregulated NF-κB and IκB-α protein levels, thereby attenuating intestinal inflammatory responses in HUA rats through modulation of the IL-17 pathway. Furthermore, ginsenoside Rg_1_ upregulated the expression of tight junction proteins ZO-1 and OCC, thereby reinforcing intestinal barrier integrity. It has been reported that beneficial bacteria such as *Bifidobacterium* and *Lactobacillus* can promote hematopoiesis and reduce tracheal inflammation through their metabolic product SCFAs, demonstrating significant implications for overall health [32]. Gut microbiota can produce SCFAs, which exhibit immunomodulatory properties. These SCFAs contribute to the restoration of intestinal barrier integrity and the regulation of gut homeostasis. Notably, deficiency in SCFAs has been implicated in the pathogenesis of various diseases, including inflammatory bowel disease (IBD) [33]. These findings collectively demonstrate that gut-resident beneficial bacteria actively generate SCFAs, exerting multifaceted positive effects on the host organism. We investigated the modulatory effects of ginsenoside Rg_1_ on the gut microbiota in HUA rats. The results demonstrated that ginsenoside Rg_1_ significantly regulated microbial diversity and richness while concurrently promoting the abundance of beneficial bacterial taxa, including *Ligilactobacillus*, *Lactobacillus*, *Ruminococcus*, and *Lachnospiraceae_NK4A136_group*. Furthermore, ginsenoside Rg_1_ intervention markedly increased the intestinal concentrations of SCFAs, including acetate, propionate, butyrate, and valerate. These SCFAs reduce inflammation and maintain intestinal barrier integrity indirectly affecting HUA.

## 5. Conclusions

In summary, ginsenoside Rg_1_ synergistically alleviated the pathological process of high-purine diet-induced HUA by remodeling the gut flora–SCFAs–barrier axis and inhibiting the IL-17/NF-κB-mediated inflammatory cascade response. This provides a theoretical basis for its clinical application and expands the research idea of intestinal flora as a new target for HUA treatment.

## Data Availability

Data are not publicly available due to privacy.
